# Cluster pattern analysis of environmental stressors and quantifying their impact on all-cause mortality in Belgium

**DOI:** 10.1186/s12889-024-18011-0

**Published:** 2024-02-21

**Authors:** Bram Vandeninden, Eva M. De Clercq, Brecht Devleesschauwer, Martina Otavova, Catherine Bouland, Christel Faes

**Affiliations:** 1 School of Public Health, Université Libre de Bruxelles, Brussels, Belgium; 2 Department of Epidemiology and Public Health, Sciensano, Brussels, Belgium; 3 Department of Chemical and Physical Health Risks, Sciensano, Brussels, Belgium; 4 Center for Demographic Research, UCLouvain, Louvain-La-Neuve, Belgium; 5 Interuniversity Institute for Biostatistics and Statistical Bioinformatics (I-BioStat), Data Science, Diepenbeek, Hasselt, Belgium; 6 Department of Translational Physiology, Infectiology and Public Health, Ghent University, Merelbeke, Belgium

**Keywords:** Spatial patterns, Clusters, Air pollution, Land cover, Noise, Public health, Ecological regression

## Abstract

Environmental stress represents an important burden on health and leads to a considerable number of diseases, hospitalisations, and excess mortality. Our study encompasses a representative sample size drawn from the Belgian population in 2016 (*n* = 11.26 million, with a focus on *n* = 11.15 million individuals). The analysis is conducted at the geographical level of statistical sectors, comprising a total of *n* = 19,794 sectors, with a subset of *n* = 18,681 sectors considered in the investigation. We integrated multiple parameters at the finest spatial level and constructed three categories of environmental stress through clustering: air pollution, noise stress and stress related to specific land-use types. We observed identifiable patterns in the spatial distribution of stressors within each cluster category. We assessed the relationship between age-standardized all-cause mortality rates (ASMR) and environmental stressors. Our research found that especially very high air pollution values in areas where traffic is the dominant local component of air pollution (ASMR + 14,8%, 95% CI: 10,4 – 19,4%) and presence of industrial land (ASMR + 14,7%, 95% CI: 9,4 – 20,2%) in the neighbourhood are associated with an increased ASMR. Cumulative exposure to multiple sources of unfavourable environmental stress (simultaneously high air pollution, high noise, presence of industrial land or proximity of primary/secondary roads and lack of green space) is associated with an increase in ASMR (ASMR + 26,9%, 95% CI: 17,1 – 36,5%).

**Supplementary Information:**

The online version contains supplementary material available at 10.1186/s12889-024-18011-0.

## Introduction

Environmental stress causes a significant burden on our human health. Environmental stress refers to various types of pollution and non-ideal environmental circumstances, such as air pollution (e.g., exposure to nitrogen dioxide (NO_2_) and particulate matter (PM_2,5_)), noise (due to road traffic, aviation, industries, etc.), lack of green spaces, industrial pollution, or exposure to non-optimal temperatures (extreme heat and extreme cold). WHO estimates that 12–13% mortality is attributable to environmental stressors, in Belgium, close to the EU-28 mean of 13% [35]. The reduction of environmental stressors exposure could prevent more than 40% of strokes, 35% of ischaemic heart disease, more than 30% of lower respiratory tract infections and 20% of cancers worldwide [24].

Air pollution is a major global cause of disease, hospitalisations, and death [7]. In 2020, 364,000 premature deaths in Europe were attributed to air pollution. The cities, with their high traffic, industry, lack of green spaces and residential areas, tend to have the most elevated levels of pollution, which can lead to higher rates of death and disease. For example, Khomenko et al. [18] found that reducing PM_2.5_ and NO_2_ concentrations in several Belgian cities to the lowest levels seen in European cities could prevent up to 7% and 6% of deaths, respectively. A literature review of PM_2.5_ and NO_2_ found an increased risk for both mortality and hospital admissions from short- and long-term exposure [1]. Noise pollution, especially from road traffic, contributes to cardiovascular disease. [11]. A 10 dB increment in road noise exposure starting at 50 dB is associated with an 8% increase in the incidence of ischemic heart disease [10]. In terms of land cover and effects on human health, access to recreational green spaces and forests can reduce mortality with an average 4% reduction in mortality per 0.1 increments in the Normalised Difference Vegetation Index (NDVI) [25]. Proximity to industrial land can worsen health and increase mortality [20]. Living near primary and secondary roads negatively affects social wellbeing, physical activity, air pollution and noise exposure. Lower IQs for children and lower cognitive abilities, worse memory and lower verbal skills for elderly living near major roadways are among the documented examples [12, 33]. The effects of proximity to agricultural land and pesticide exposure are less clear, with some studies finding decreased mortality [27] and others finding increased risks for specific diseases such as Parkinson’s from glyphosate exposure [6].

Previous studies primarily considered the singular effect of individual environmental stressors on the country level. Nevertheless, only a few studies considered sub-national spatial patterns or cumulative effects of multiple environmental stressors exposure on health. While there is limited evidence indicating environmental stressors can work together to have synergistic effects on human health, more research is needed to understand those interactions and the mechanisms behind them (EEA, Agency and [7].

In this study, we performed a cluster analysis including, simultaneously, multiple environmental stressors to consider non-linear relationships between the environmental stressors, mortality, and socio-economic factors. It allows us to analyse the spatial pattern of environmental stress in Belgium, identifying patterns between those elements with more coherence. This paper aims to unravel the geographical patterns of environmental stressors in Belgium. We consider air pollution, exposure to agricultural land and associated pesticides, the presence of industrial sites, busy roads and related noise, and the lack of green space.

In the pursuit of an improved understanding of environmental mortality determinants, we examine the relationship between cluster patterns of detected environmental stressors and mortality using an ecological regression model and relevant literature (e.g., meta-analyses of individual-level cohort studies).

## Methodology

The analysis is performed at the statistical sector level. This is the smallest administrative subdivision of Belgium. There are 19 794 statistical sectors in Belgium (2020). Their mean population size is 550 inhabitants. The geographical size varies depending on population density. In areas with the highest population density, statistical sectors tend to be very small, while in less populated rural areas, statistical sectors tend to be larger.

### Study setting and design

Belgium is a country in Europe with a population of 11.6 million inhabitants (2021). 31% of the population lives in cities, while 56% of the population lives in towns and suburbs and 13% of the population in rural areas according to the Eurostat classification for the year 2021 [8].

The health and environmental policies in Belgium are in part organised at the federal level, and in part on the level of the 3 administrative regions Brussels, Flanders, and Wallonia.

Statistical sectors are the smallest administrative subdivision in Belgium. There are 19 794 statistical sectors in Belgium with on average 500 inhabitants living in one statistical sector. In terms of geographical area, statistical sectors are smaller in densely populated urban areas and larger in rural areas.

For the clustering analysis, all 19 794 statistical sectors within Belgium are used. For this mortality calculation, all 19 794 statistical sectors in Belgium are used. For the ecological regression analysis, data with missing values are removed. Data with missing values include statistical sectors which less than 5 deaths over the considered period, which are removed from the dataset for privacy concerns. In the final analysis, 18 681 statistical sectors (94.4% of sectors, 99.2% of population) remained available for inclusion.

### Mortality data

Observed mortality per statistical sector per age group of 10 years (0-9 year, 10–19 year, 20–29 years, etc.) aggregated for the period 2012–2016 were obtained via the DEMOBEL database, part of the Causineq dataset, also related to Statbel.

### Exposure assessment

Data on environmental stressors include high-resolution (10 × 10 m) annual air pollution maps of PM_2.5_, NO_2_, BC and O_3_ from the year 2018 (derived from the open-data endpoint https://www.irceline.be/en/documentation/open-data/open-data?set_language=en), High resolution (100 × 100 m) land cover data from CORINE land cover for the year 2018 (derived from https://land.copernicus.eu/pan-european/corine-land-cover/clc2018), data on primary and secondary roads (derived from Statbel, https://statbel.fgov.be/), vector data on noise segments for Flanders and Wallonia 2016–2017 (obtained via regional government data) and noise raster data for Brussels (obtained via regional government data, e.g. “omgeving Vlaanderen”, “Leefmilieu Brussels”, “Geoportail Wallonie”).

Environmental stressors data are post-processed (in a GIS environment using ArcMap) into mean values per statistical sector. For Air Pollution, mean annual PM_2.5_, NO_2_, BC and O_3_ concentrations per statistical sector for air pollution are calculated (2018) derived from original 10 × 10 m resolution model raster maps for those air pollutants. The original data is a Land-Use-Regression derived air pollution model where local and regional emissions are added to the model, which together is named the RIO-IFDM model chain [15, 19]. In recent years, an improved version of the model was launched, named ATMO-Street. The building stones of this model remain similar compared to the model described above. However, in addition, this model takes into account concentration peaks in Street Canyons. Validation showed the model passes the European FAIRMODE quality criteria, deeming the model chain suitable for policy support [14]. All concentrations are expressed in µg/m^3^. In our analysis, we used the improved ATMO-street model.

For Land Cover, maps from 2018 from CORINE Land Cover are used. Those data are based on remote sensing images using the Sentinel-2 and Landsat-8 satellites. In addition, a GIS-dataset on primary and secondary roads, based on OpenStreetMaps combined with the official road type classifications was used. The spatial resolution of the data from this source are at least 100 m. In our post processing of the data, the mean fraction of agriculture, industry, primary and secondary roads, green and forested areas per statistical sector are calculated. The mean fraction is the percentage of the surface of the considered statistical sector that is characterised by this type of land cover. For example, a statistical sector with a surface area of 2.1km^2^, characterised by 0.2km^2^ of primary and secondary roads, 0.8km^2^ of agriculture, 0.4km^2^ of residential houses and 0.7km^2^ of industry area has an agriculture fraction of 0.38, an industry fraction of 0.33 and a primary & secondary road fraction of 0.10. Validation of the CLC2018 land cover data showed average accuracies of > 92.0%, passing the recommended threshold of 85% [29].

For noise, the percentage of the statistical sector characterised by road, railway, and airplane noise between 55 and 70 dB and above 70 dB is calculated based on original vector files of noise for the Flanders and Wallonia region, which contained noise segments for each category for locations where the threshold of 55 dB is exceeded and calculated based on a raster file containing noise values at high 100-m resolution level for the Brussels area. The Brussels and Flanders data are yearly averages for the year 2016 while the Wallonia data is a yearly average for the year 2017. Those data are post processed into the same data format of each region, containing per statistical sector the area percentage of the statistical sector exceeding respectively 55 and 70 dB noise for the Lden indicator. All noise data are developed in accordance with the CNOSSOS-EU noise guidelines [17].

The input data described here are subsequently used for hierarchical clustering via the Ward Algorithm, which for each category of environmental data, namely air pollution, land cover and noise, classified the statistical sectors into clusters. The section “statistical analysis” elaborates on this further.

### Covariate data

Co-variate data and confounding factors are pivotal in health-environment relationships. Socio-economic disparities, like low education and employment, often align with increased environmental stress, such as elevated air pollution or reduced green spaces [2].

In our study, Socio-economic were aggregated at the level of the statistical sector. For this we used an index of multiple Deprivation (IMD) for Belgium [22], consisting out of a weighted average of multiple deprivation domains (health, income, education, crime, employment and housing). The domains in the IMD are based on the aggregation of census data indicating a form of deprivation from the year 2011. For example, the housing domain contains indicators as the proportion of individuals living in dwellings, being tenants, having a living area < 35m^2^, without central heating, without insolation, without kitchen, without toilet, without bathroom and without internet. More information on the IMD, including its domains, parameters, weights and calculation methods can be found in the scientific publication where the index is discussed [22].

Further, age-specific population data for the year 2016 were obtained via Statbel. This contains the number of people per statistical sector per age group of 10 years (0–9 years, 10–19 years, 20–29 years, etc.)

For the data layer of the regions, the 19 794 statistical sectors are classified into 3 categories: Flanders, Wallonia, and Brussels, corresponding to those administrative regions. As important parts of health and environmental policies are a responsibility of the regions rather than the national government, different policies in those regions may have led to different strategies related to confounding factors such as tobacco and alcohol, for example in the domain of health prevention. For example, adding the regions as a variable in the model reduces the influence of the confounding factor smoking. The factor smoking explains around 19% of mortality in Belgium [9], with important spatial variations, namely attributable mortality ranging from 10 to 47% between communities [23]. Smoking prevalence varies geographically, with lower rates in Flanders, higher rates in many Walloon municipalities, and an intermediate position in the Brussels-Capital Region. Urban areas, with higher air pollution and noise, have younger and more educated residents, often exhibiting lower smoking prevalence, potentially mitigating environmental-related mortality [26],Teughels et al., [28]. In 2018, daily smoking prevalence was 29% for those without an educational degree and 9% for tertiary education holders.

### Statistical analysis

#### Clustering

The output of the mapping of environmental stressors per statistical sector was used as an input for the hierarchical clustering. The principle of the hierarchical clustering analysis aims to minimise variance between statistical sectors within a cluster and to maximise variance between statistical sectors belonging to different clusters. The Ward algorithm of the hclust package in R (versions 4.1.2 and 4.1.3) is used to conduct the cluster analysis [32]. Statistical sectors are merged into clusters, thereby minimizing information loss.

Three separate environmental cluster groups are created: air pollution clusters, noise clusters and land cover clusters. The air pollution clusters are based on the pollutants: NO_2_, PM_2.5_, BC and O_3_. For the noise cluster, road, rail and aeroplane noise between 55 and 70 dB and above 70 dB are considered. For the land cover clusters, the following variables are included: industry, agriculture, forests and green spaces, primary and secondary roads.

In addition, a fourth cluster group consists out of the housing, education and crime dimension of the Index of Multiple Deprivation. The Health and Employment domain were excluded from the analysis, as they contain respectively the age-standardized mortality rate and the percentage of people living with a disability / on long-term sick leave, which kind of outcomes are part of our independent variable in the ecological regression model in the next step. Cluster characteristics for the socio-economic cluster are displayed in Additional file 1: Figure S1.

To determine the number of clusters, a cut-off value of explaining at least 95% of the variance for all cluster groups was chosen. Based on this, the number of five clusters is chosen for all cluster groups.

#### Mortality attributable to the air pollution clusters based on Population Attributable Fraction (PAF)

The relative risk (RR) between an increase in air pollution concentrations and age-standardized mortality rate is well-established in existing studies and meta-analysis of cohort studies on the individual level. The ELAPSE meta-analysis [5, 13] is the most recent elaborate meta-analysis providing RR estimates for PM_2.5_ (1.118 [1.060 – 1.179] per 10 µg/m^3^ increase and NO_2_ (1.045 [1.026 – 1.065] per 10 µg/m^3^ increase). Due to the inclusion of more recent meta-analysis with better exposure assessment methodologies such as higher resolution and a focus on the Europe Region, the ELAPSE meta-review has a comparative advantage over older meta-analysis available. To calculate the PAF, the fraction of mortality attributable to the different air pollutants, based on the following formula:

$$PAF=\frac{p.\left(RR-1\right)}{1+ p.\left( RR-1\right)}$$

in which $p$ represents the prevalence of the exposure and $RR$ is the relative risk of the exposure.

The RR is recalled from the default unit per 10 µg/m.^3^ increase to the relevant exposure unit, using

$$RRexposure=exp((ln(RR10)/10)*(CON))$$

In which RR10 is the RR for an increase in 10 µg/m^3^ and CON is the actual mean concentration of the statistical sector of the air pollutant under consideration. The confidence intervals of our analysis originate from conducting a Monte Carlo Simulation assuming a triangular distribution.

#### Ecological regression model

The relations between the exposure to environmental stressors through the clusters and total mortality are determined using a generalised linear regression model at the ecological level of the statistical sector. We improved the existing ecological studies by using smaller administrative units, thereby reducing the heterogeneity within the areas, resulting in finer ecological exposure.

A negative binomial model was used as our epidemiological data showed a Poisson distribution with a large amount of overdispersion. A 26% drop in Bayesian Information Criterion (BIC) objectified our choice of a negative binomial model over a Poisson model. The response variable is the number of deaths per statistical sector. Independent variables include cluster groups of air pollution, noise stress and land cover, socio-economic and health variables (more details below). The expected deaths per statistical sector were taken as an offset, to account for the population size and age-distribution in the statistical sector. The ASMR is calculated from the indirect standardised rate with the whole of Belgium as the standard population.

The baseline model included the Air Pollution, Land Cover, and Noise clusters as covariates. A second version of the model also contains a cluster with the housing, crime, education, and income dimensions of the Index of Multiplde Deprivation (IMD). Careful interpretation of the results is necessary as multicollinearity can result in type I and type II errors [16].

We also investigated if the model fit improved adding the regions correcting for different lifestyles which have major impact on the most important confounding factors such as smoking. The model fit improved considerably, resulting in the inclusion of the regions in the model (Flanders, Brussels, and Wallonia).

In the dataset for the negative binomial regression model, we only used the statistical sectors for which no missing values exist for any of the variables to ensure statistic consistency of the analyses (17 434 statistical sectors). The removed statistical sectors in this procedure are dominantly small statistical sectors for which some of the variable values are not available due to privacy concerns.

This has led to the following regression model:

$${Y}_{i}\sim NegBin({E}_{i}R{R}_{i},k)$$

$$log\left(R{R}_{i}\right)= \sum_{k}{\alpha }_{k}{Reg}_{i}+\sum_{k}{\beta }_{k}Ai{r}_{i}+\sum_{k}{\gamma }_{k}Nois{e}_{i}+\sum_{k}{\delta }_{k}Landcove{r}_{i}+\sum_{k}{\delta }_{k}{socio-economic}_{i}$$

with ${Y}_{i}$ the total number of deaths occurring in each statistical sector over the considered period, ${E}_{i}$ the expected number of deaths expected to occur in each statistical sector over the considered period, $R{R}_{i}$ the incidence risk ratio to be estimated and $k$ and overdispersion parameters. In the regression model, ${Reg}_{i}$ corresponds to the Belgian regions (Flanders, Wallonia and Brussels), $Ai{r}_{i}$ corresponds to the five air pollution clusters (denoted as clusters A to E), and similarly $Nois{e}_{i}$ and $Landcove{r}_{i}$ correspond to the five noise stress and five land cover stress clusters. Finally, $Socio-{economic}_{i}$ corresponds to the five socio-economic clusters based on the income, education, crime and housing domain of the IMD.

In 2nd model version, we replaced the clusters of environmental stress by an index of cumulative exposure. We create three categories depending on the favourability of the conditions of environmental stress. The three categories are “HIGH” (the combination of clusters for which we expect based on literature the most unfavourable health outcomes: statistical sectors belonging to the cluster with very high air pollution values + high noise values + land cover characterised by considerable amounts of industrial land or high fractions of primary and secondary roads), “LOW” (expected most favourable environmental conditions for health: statistical sectors characterised by the combination of the cluster with the lowest air pollution + cluster with no noise exceedance + cluster with a high fraction of green spaces) and “MEDIUM” (everything in between). To compare the socio-economic variables a similar level, the socio-economic cluster based on the aforementioned dimension of the IMD was added to the model. the. This has led to the following regression model:

$${Y}_{i}\sim NegBin({E}_{i}R{R}_{i},k)$$

$$log\left(R{R}_{i}\right)= \sum_{k}{\alpha }_{k}{Reg}_{i}+\sum_{k}{\beta }_{k}Cum{E}_{i}+\sum_{k}{\gamma }_{k}Socio-Economi{c}_{i}$$

With $Cum{E}_{i}$ a categorical classification of the amount of cumulative environmental stress and $Socio-Economic_{i}$ the index of multiple deprivation (income, education, crime and housing dimension).

Importantly, and this is true for all models: our model's purpose is not centered around predictive capabilities; rather, it serves as a tool for studying associations.

## Results

### Spatial patterns of environmental stressors through clustering

#### Air pollution

**Fig. 1:**
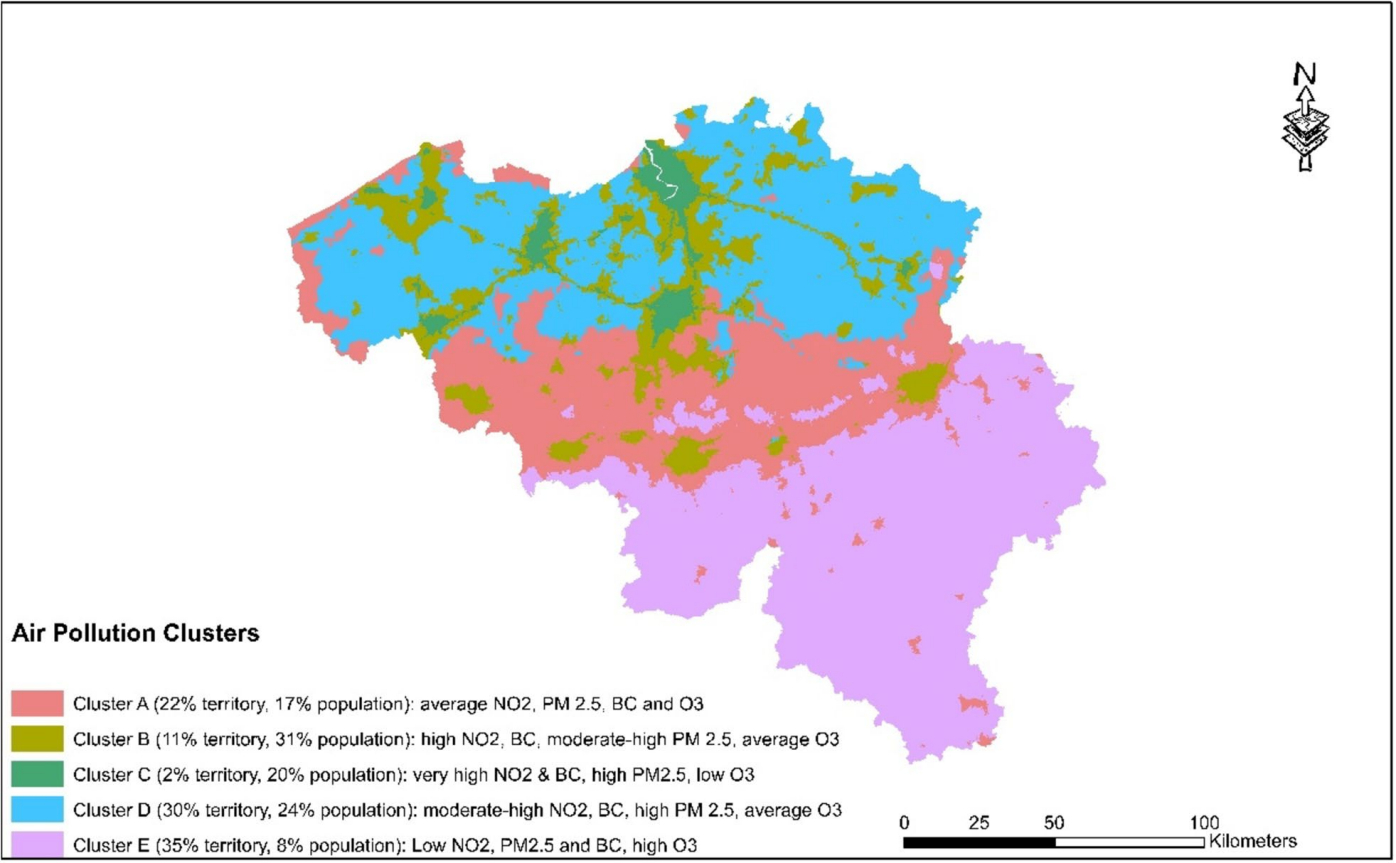
Air pollution clusters in Belgium

**Fig. 2:**
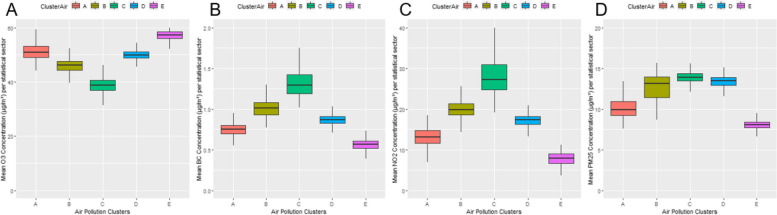
Descriptive statistics (population-weighted boxplots) of the respective air pollution clusters in Belgium

Five clusters of air pollution have been identified in Belgium, as represented in Fig. 1. Descriptive statistics of the clusters can be found in Fig. 2. Cluster E, located in southern Wallonia, has the lowest concentrations of NO_2_, BC and PM_2.5_, but the highest concentrations of O_3_. It covers 35% of the territory but only 8% of the population. Cluster A, which encompasses parts of Walloon Brabant, West Flanders, Hainaut, Liège, and Limburg, has intermediate pollutant levels and includes both agricultural and urban areas. Clusters B and D, which include cities in Wallonia and suburban areas in Flanders with heavy traffic, have moderate to high pollutant levels, and intermediate ozone levels. Notably, Cluster C has the highest values of NO_2_, BC and PM_2.5_ and while it covers only 2% of the territory, it has 20% of the Belgian population. All clusters exceed the PM_2.5_ guideline of 5 µg/m^3^ set by the WHO. Cluster E is the only cluster that meets the WHO guideline for NO_2_ of < 10 µg/m^3^.

#### Land cover

**Fig. 3:**
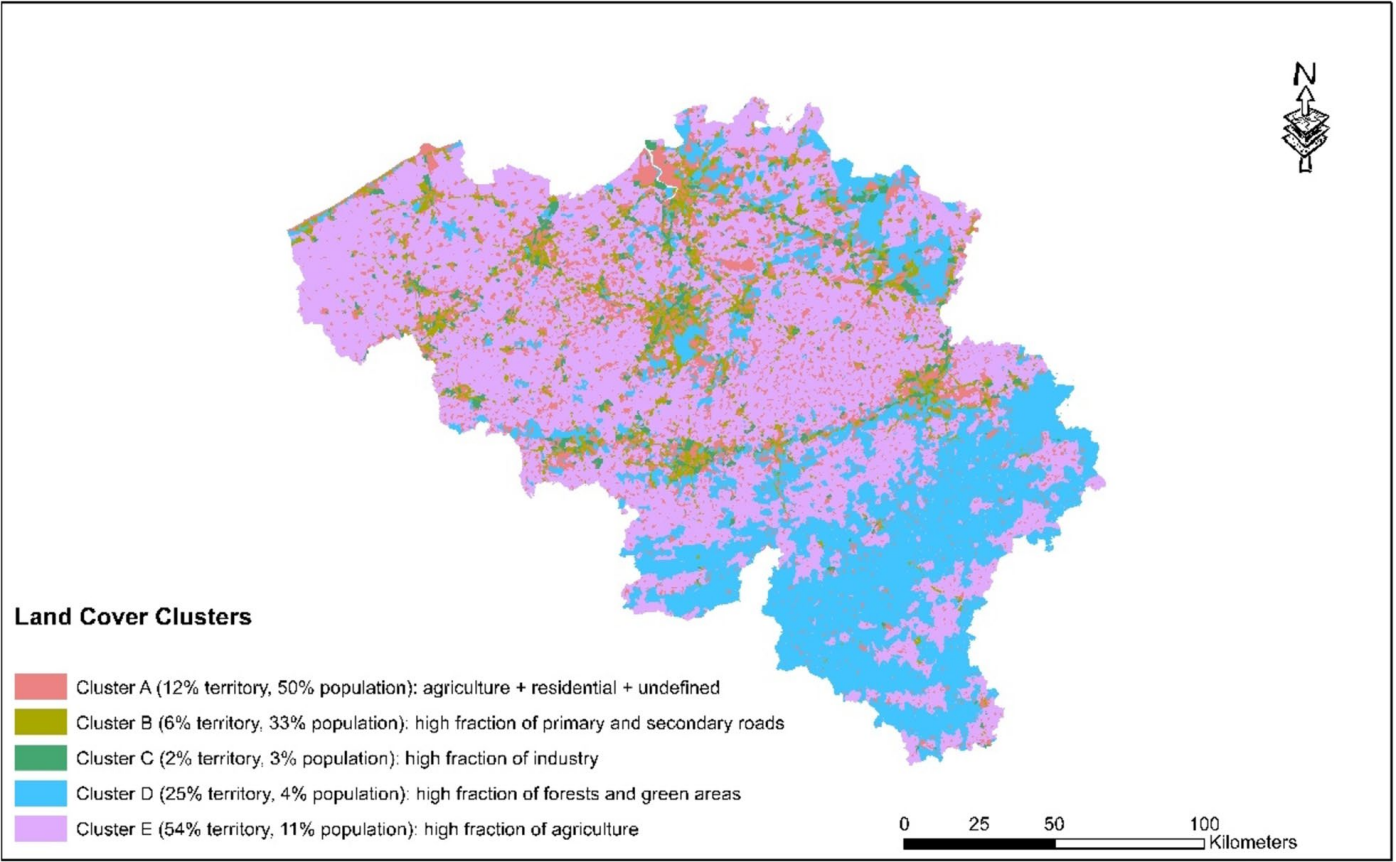
Descriptive statistics (population-weighted boxplots) of the respective Land Cover Clusters in Belgium

**Fig. 4:**
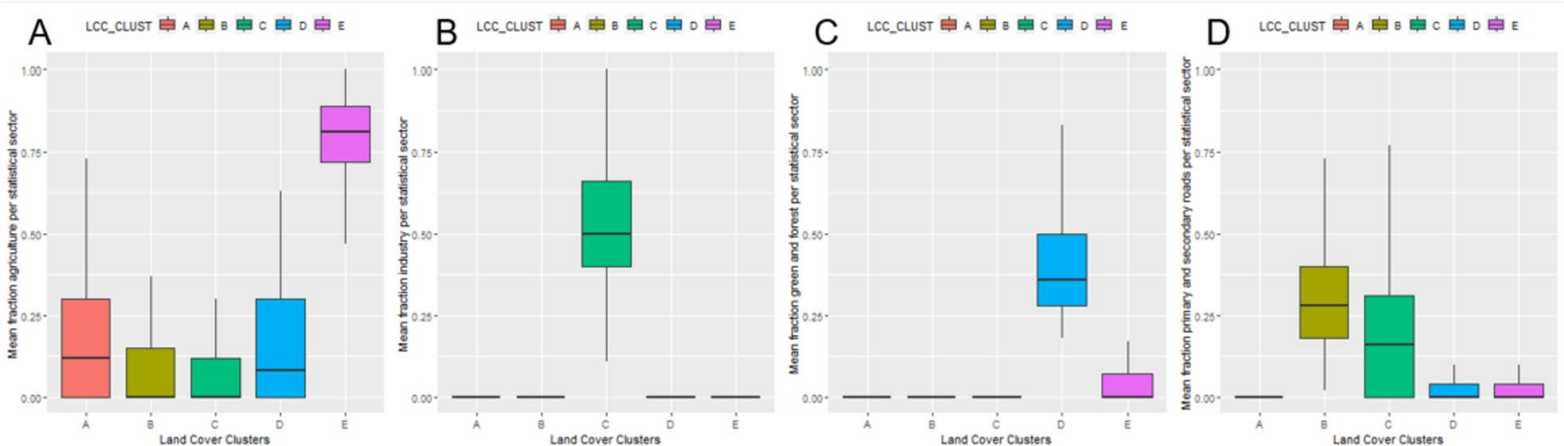
Descriptive statistics (population-weighted boxplots) of the respective Noise Clusters in Belgium

Five land cover clusters have been identified (Fig. 3). Descriptive statistics are shown in Fig. 4. Cluster A, where 50% of the population lives, is primarily a residential cluster characterised by moderate fractions agricultural land, with a lack of industrial areas, green spaces, and limited presence of primary and secondary roads. Cluster B (33% of population, 6% of territory), primarily found in middle-sized and large cities such as Brussels, Antwerp, Liège, Charleroi and Ghent is characterised by low agricultural land, a high presence of primary and secondary roads, and an absence of green areas. Cluster C (only 3% of population and 2% of territory) is characterised by high fractions of industrial land (50–75% of the area) and moderate presence (0–25%) of primary and secondary roads. Cluster D (4% of population, 25% of territory) is primarily found in forested areas of the Ardennes and some parts of Limburg and is covered by abundant forests and recreational green spaces with 50% of the surface in the statistical sectors belonging to this cluster.

#### Noise

**Fig. 5:**
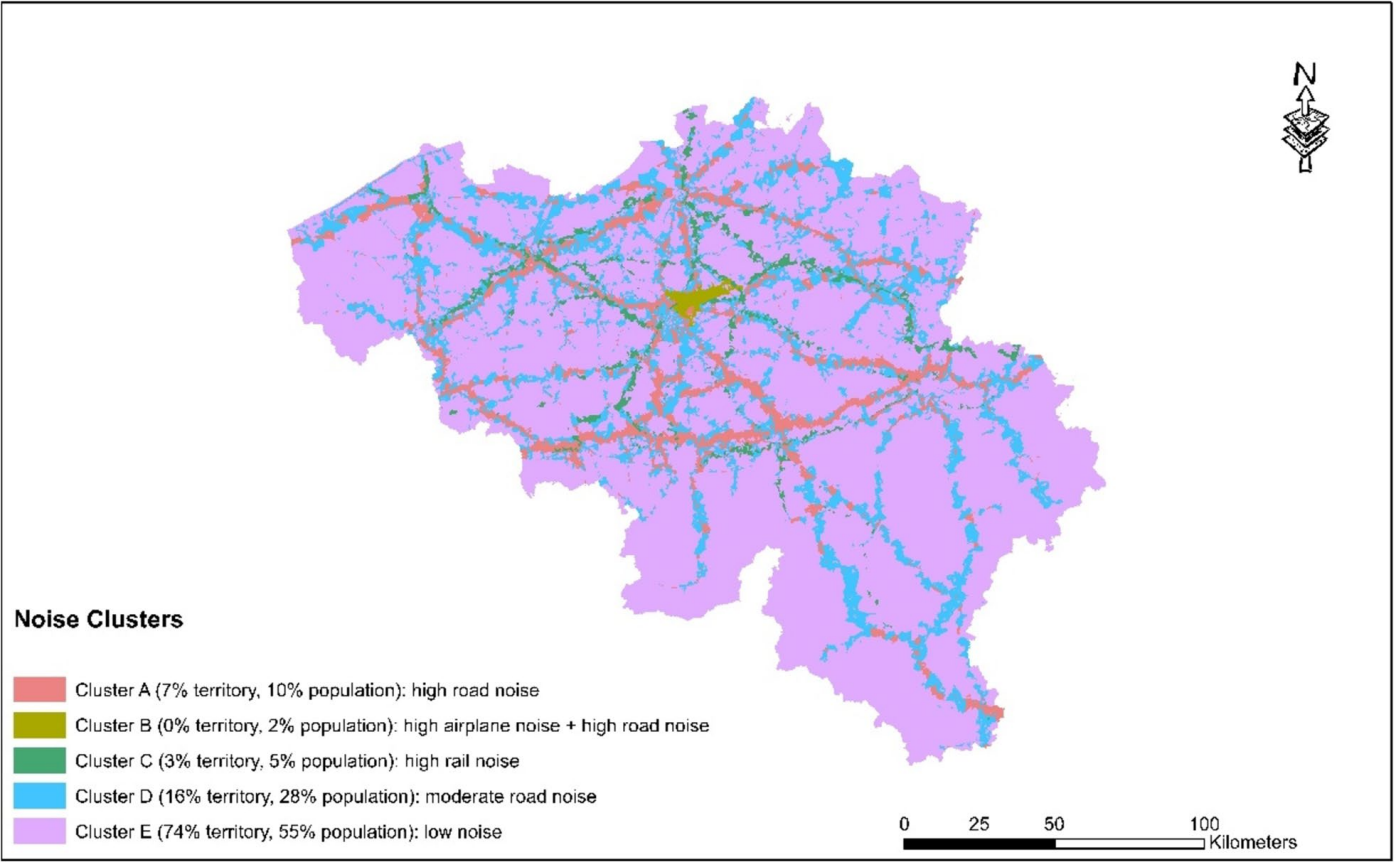
Noise Stress clusters in Belgium

**Fig. 6:**
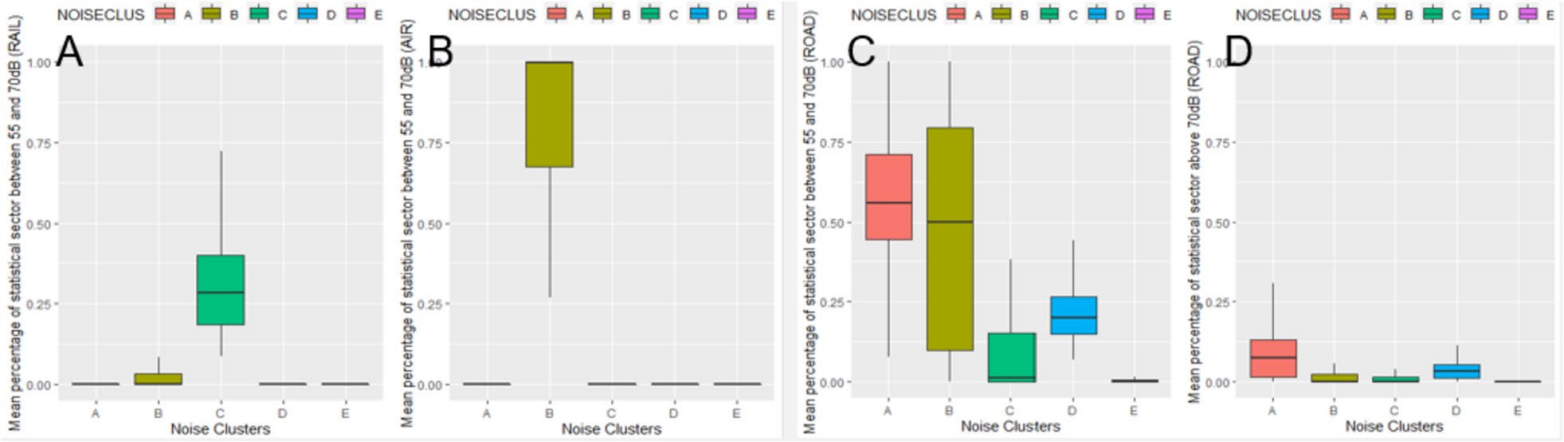
Descriptive statistics of the noise clusters in Belgium

Five noise clusters have been identified in Belgium (Fig. 5). Descriptive statistics are shown in Fig. 6. According to the EU’s Environmental Noise Directive, the threshold for excess exposure, defined as Lden, is 55 dB. Lden, measured in decibels (dB), indicates an average level during the day, evening, and night. Cluster A is characterised by high levels of road noise, with over 50% of the area exceeding > 55 dB(< 70 dB) Lden and over 5% exceeding 70 dB Lden. Cluster B is characterised by a combination of high levels of aeroplane noise and road noise, with over 50% of the area exceeding > 55 dB(< 70 dB) Lden from both sources. Cluster C is characterised by high levels of railway noise, with 25% of the area exceeding > 55 dB (< 70 dB) Lden. Cluster D is characterised by high levels of road noise, with 20–25% of the area exceeding > 55 dB Lden. Cluster E does not exceed the 55 dB Lden threshold for road, railway, or airplane noise. This cluster covers 74% of the territory and 55% of the population lives in this cluster without any noise exceedances. 45% of the population lives in one of the other clusters where there are noise exceedances for at least one source of noise.

#### Cumulative environmental stress

**Fig. 7:**
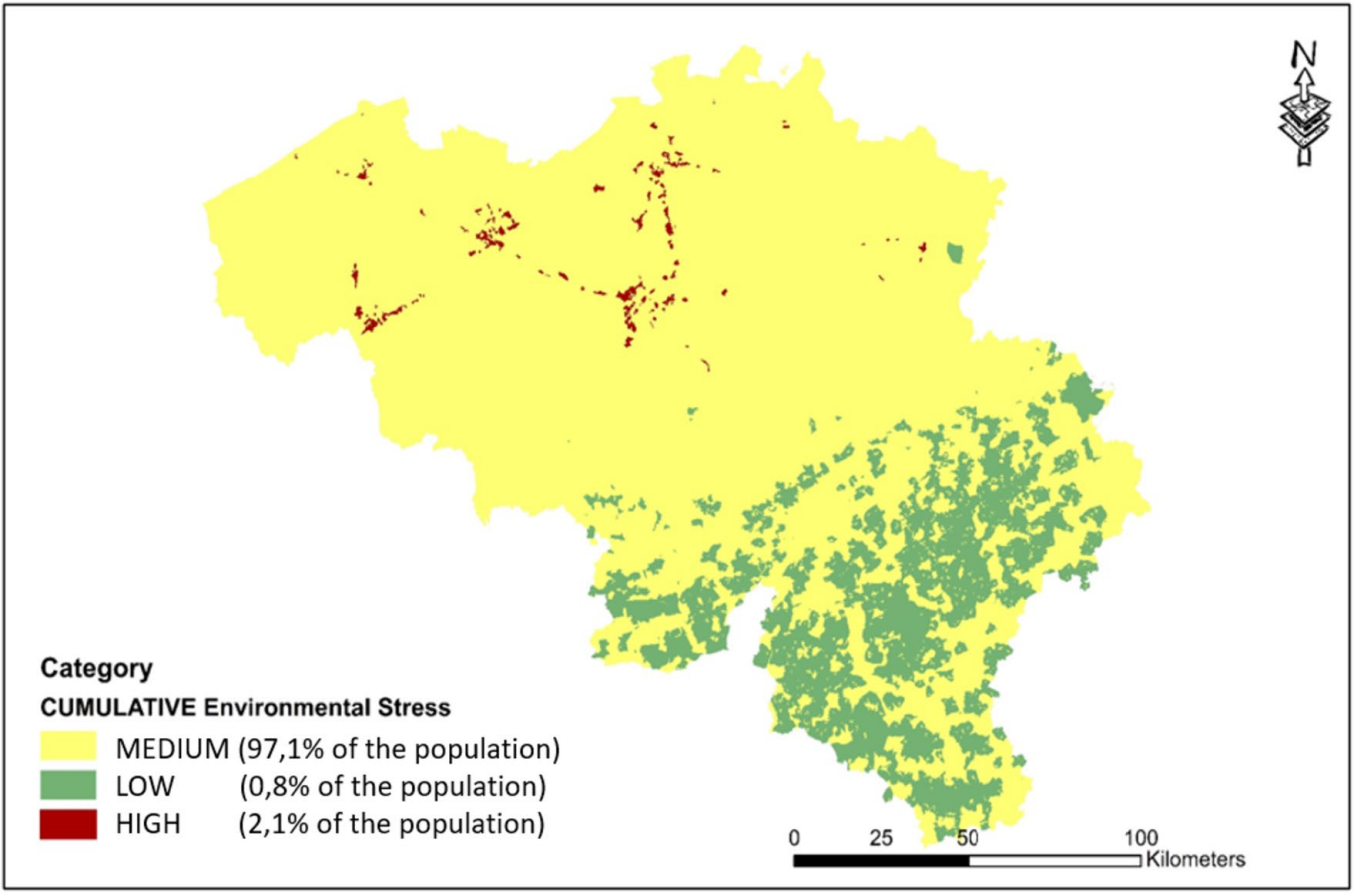
Cumulative exposure to average vs. very high simultaneous (elevated air pollution + high industry/roads + high noise) and very low simultaneous (abundant green space, low air pollution and low noise) environmental stress: geographical map of Belgium

Figure 7 illustrates the cluster categories used as input for the cumulative regression model where simultaneous exposure to multiple favourable and unfavourable conditions is grouped together. We observe that exposure to simultaneous favourable conditions such as co-exposure to sufficient residential green space, low levels of noise and air pollution, mainly occurs in the southern part of Belgium where the most hotspots of unfavourable simultaneous exposure to multiple stressors happens mainly in some smaller spots in the northern part of Belgium. However, the limited geographical extent of the latter category, 2.1% of the population, around 250 000 residents, live in this category, much more than the 0,8% of the population living in the much more extensive geographical area with simultaneous favourable environmental stress conditions.

### Environmental stress and mortality

#### Mortality attributable to the air pollution clusters

Air pollution clusters C and E have the highest and lowest PAF values for both NO_2_ and PM_2.5_, respectively. Cluster C specifically has an 11,9% [7,2 – 16,9%] and 14,8% [9,1 – 20,2%] PAF for NO_2_ and PM_2.5_ respectively, meanwhile cluster E has the lowest, with other clusters falling in between (Table 1). The diversity of PAFs for NO_2_ is substantial, ranging from mean estimates of around 3% in cluster E to 12% in cluster C, a four-fold difference. The difference in PAFs for PM_2.5_, however, is less pronounced, with cluster C having a PAF that is less than double of cluster E.

**Table 1:** Mean air pollutant values and PAF all-cause mortality for the different air pollution clusters

Air pollution Clusters	Mean PM_2.5_ Value	Mean NO_2_-Value	PAF Mortality to PM_2.5_ RR 1.118 per 10 µg/m^3^ [1.06 – 1.179]	PAF Mortality to NO_2_ RR 1.045 per 10 µg/m^3^ [1.026 – 1.065]
Air pollution Cluster A	10.1 ± 1.1 µg/m^3^	12.9 ± 2.0 µg/m^3^	11,00% [6,48%—15,63%]	5,78% [3,45%—8,27%]
Air Pollution Cluster B	12.7 ± 1.5 µg/m^3^	19.9 ± 2.4 µg/m^3^	13,75% [8,05%—19,36%]	8,66% [5,40%—12,16%]
Air Pollution Cluster C	14.0 ± 0.7 µg/m^3^	27.5 ± 4.2 µg/m^3^	14,80% [9,12%—20,16%]	11,93% [7,23%—16,91%]
Air Pollution Cluster D	13.4 ± 0.7 µg/m^3^	16.9 ± 1.5 µg/m^3^	14,15% [8,82%—19,37%]	7,49% [4,72%—10,51%]
Air Pollution Cluster E	8.0 ± 0.5 µg/m^3^	7.2 ± 1.6 µg/m^3^	8,70% [5,27%—12,11%]	3,30% [1,84%—4,94%]

#### Negative binomial regression model

**Fig. 8:**
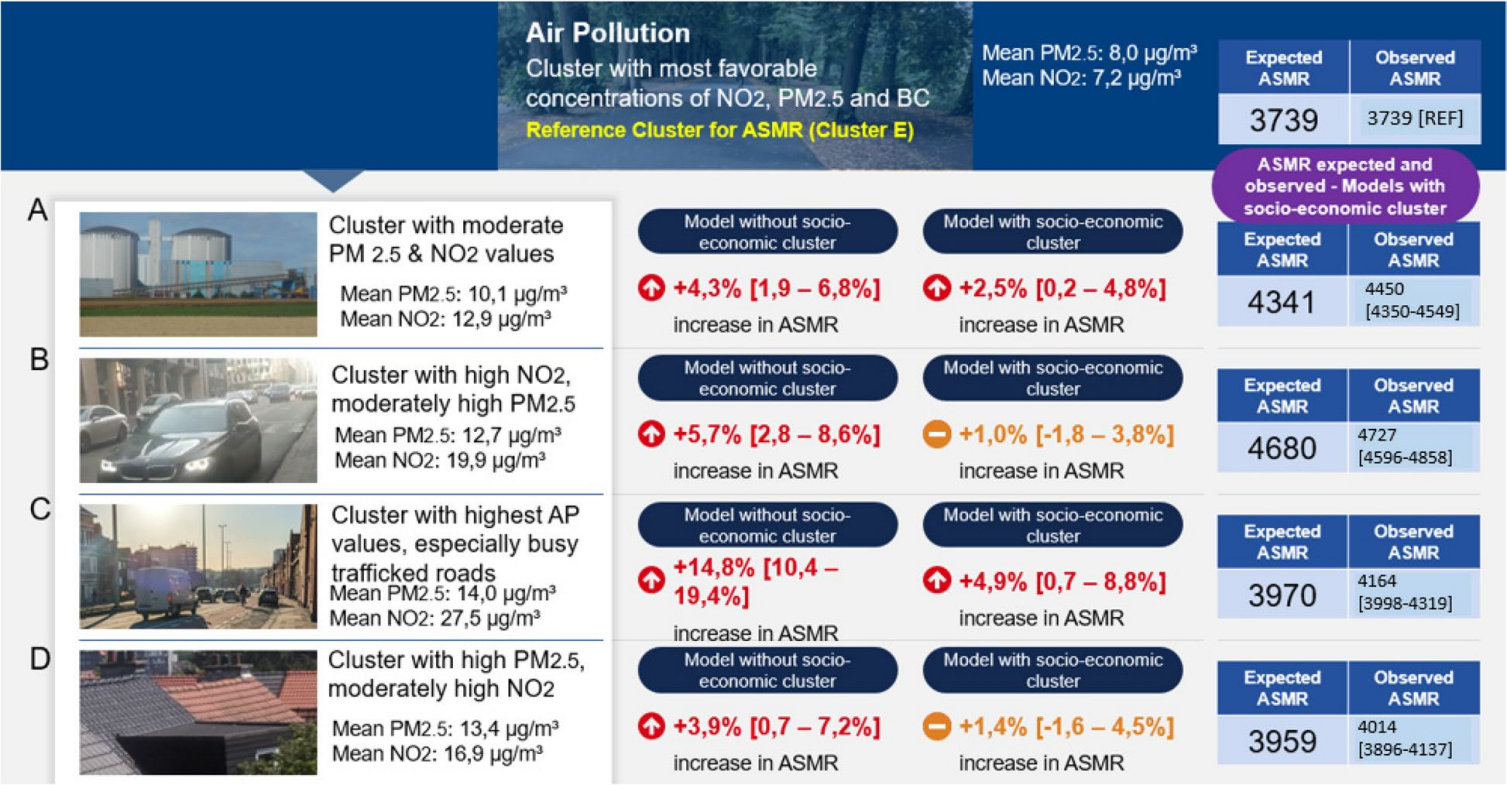
Summary outputs of the regression model investigating relation between ASMR and the clusters of environmental stress. Output estimates for the air pollution clusters. Own Photographs ©Bram Vandeninden

**Fig. 9:**
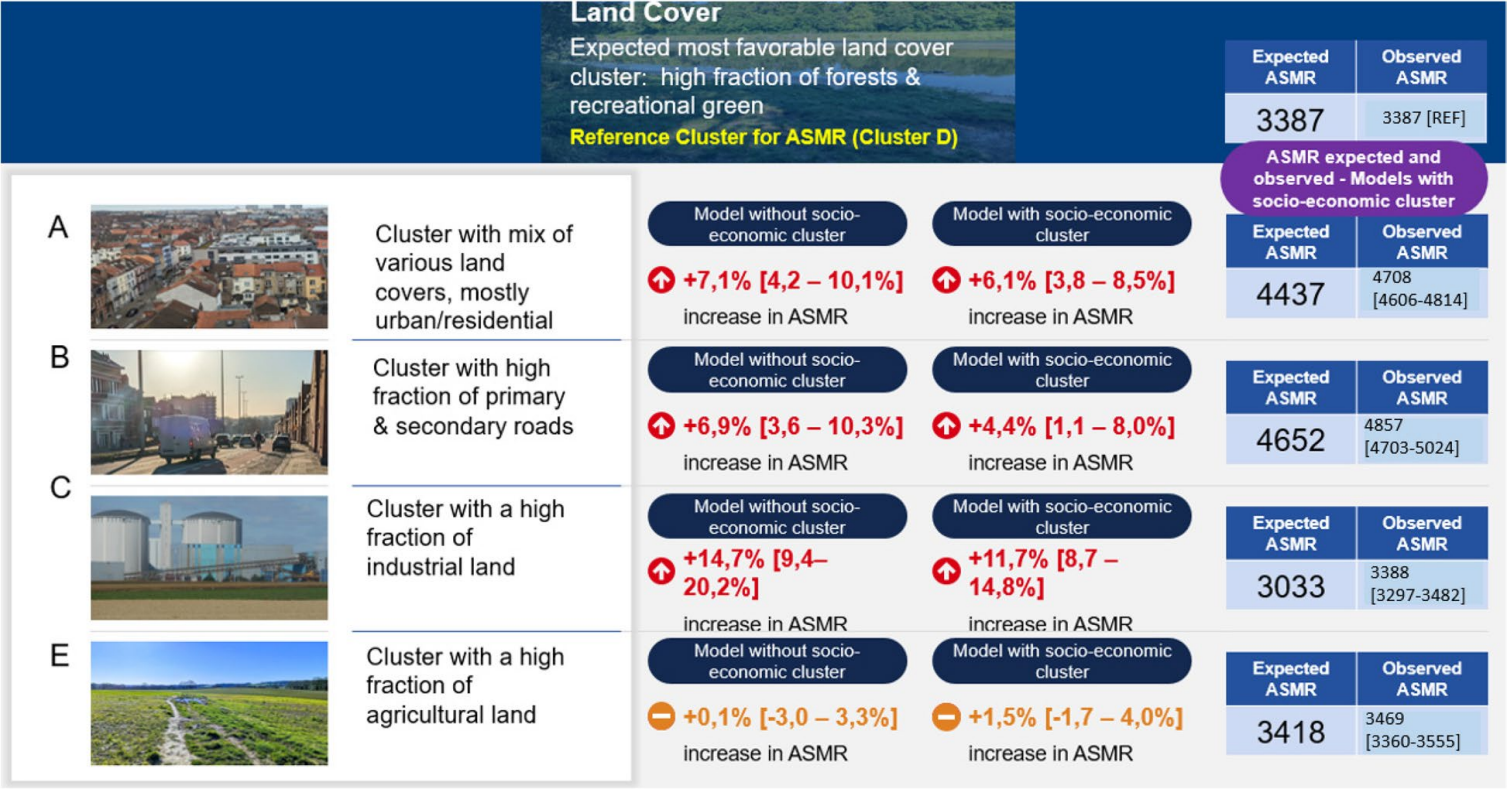
Summary outputs of the regression model investigating relation between ASMR and the clusters of environmental stress. Output estimates for the land cover clusters. Own Photographs ©Bram Vandeninden

**Fig. 10:**
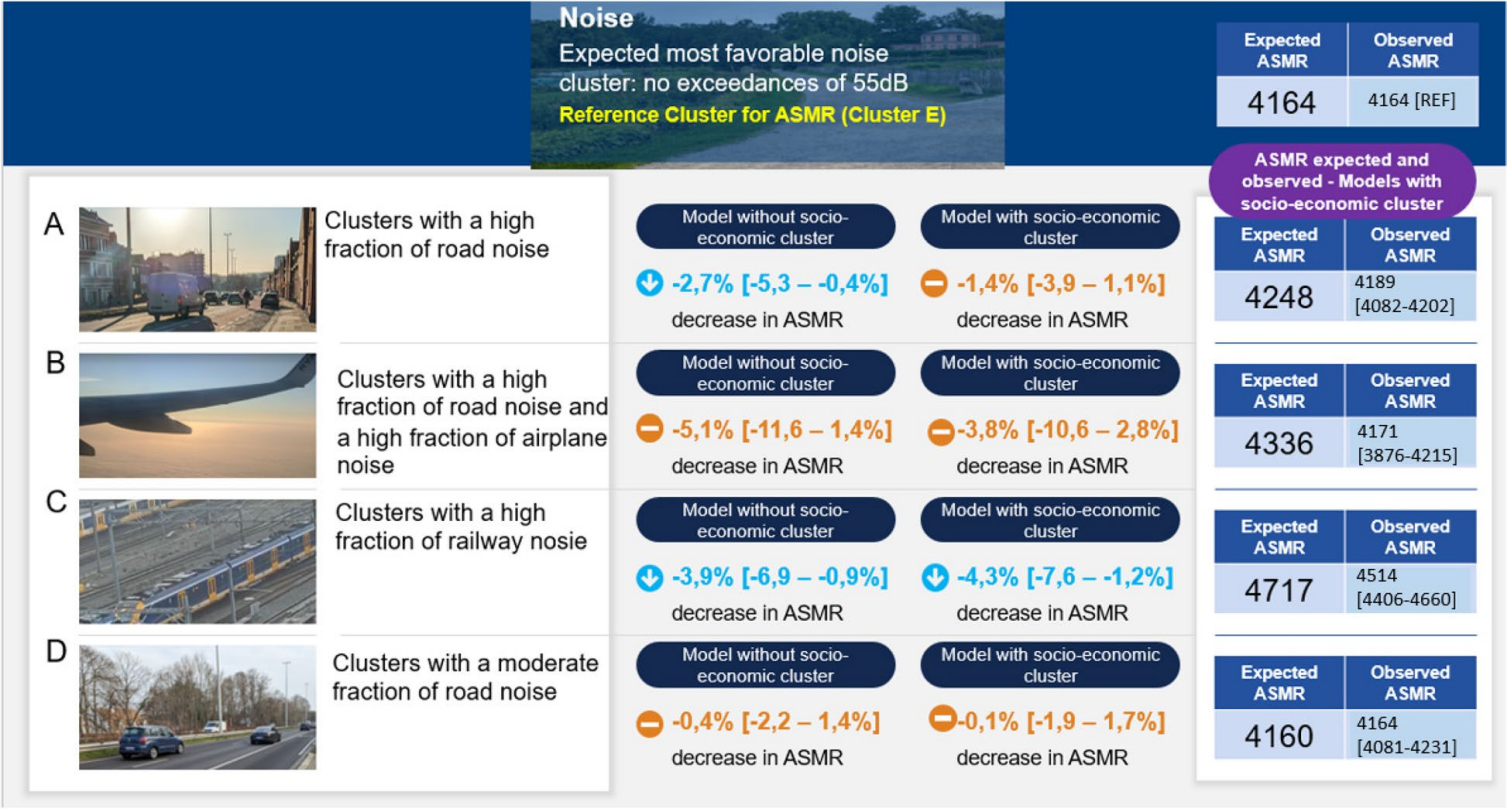
Summary outputs of the regression model investigating relation between ASMR and the clusters of environmental stress. Output estimates for the noise clusters. Own Photographs ©Bram Vandeninden

The Figs. 8, 9 and 10 summarise the results of the model considering all clusters of environmental stress (air pollution clusters, land cover clusters and noise clusters). We display both the increased/decreased percentage in ASMR for the model without socio-economic variables and model with socio-economic variables. For all cluster groups, the cluster with the most favourable environmental stress conditions (lowest mortality to be expected based on existing evidence in literature), is taken as a reference and the AMSR – for the model version including socio-economic variables is compared for the other clusters compared to this reference cluster. The more detailed row outputs for the regression models can be found in the appendices of this paper (Additional file 1: Table S1, S2, S3 & S4).

All air pollution clusters (A, B, C, and D) show an increased ASMR compared to reference cluster E with the lowest mortality, with a sharp increase in ASMR of 14.8% [95% CI: + 10,4—+ 19,4%] for Air Pollution cluster C with the most elevated AP levels of BC, PM_2.5_ and NO_2_ in the model without socio-economic variables. After correcting for the socio-economic variables, the increase in ASMR diminishes however remains significant for the residential cluster A and the cluster with high traffic-associated air pollution values cluster C who after adjustment have + 2,5% [+ 0,2%—+ 4,8%) and + 4,9% [+ 0,7—+ 8,8%] higher observed ASMR compared to the expected ASMR respectively.

For land cover, we detect no significant difference in ASMR between the most favourable green/forested reference cluster (D) and cluster E dominated by agriculture. The residential cluster (A) and cluster B with a high fraction of primary and secondary roads show a moderately increased ASMR. The cluster covered by industrial land shows the highest mortality with an increase of 14,7% [+ 9,4—+ 20,2%] in ASMR compared to the reference cluster in the model without adjustment for socio-economic factors. Adjustments for socio-economic factors has a small effect. For the industrial cluster, the increase in ASMR remains very high with + 11,7%

For the noise clusters, no increased mortality compared to the most favourable reference cluster (no noise exceedances) were detected. Most estimates tend to find a protective effect of noise on health, however the estimates are not significant. Except for cluster C (railway noise) where noise showed a significant decrease in ASMR of -3,9 [-6,9—-0,9%]. Presence or absence of adjustment for socio-economic factors had little impact in the noise clusters.

**Fig. 11:**
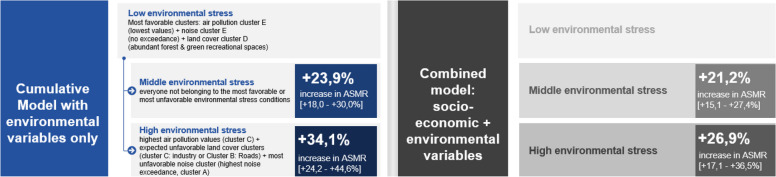
Cumulative Regression model including categories of cumulative environmental stress and cumulative socio-economic deprivation

For the cumulative exposure model, comparing three classes of total environmental stress represented by cluster combinations, the ASMR was increased by 34,1% [95% CI: 24,2 – 44,6%] in the areas with a high cumulative dose of environmental stress compared to the cluster exposure combinations with the most favourable environmental stress conditions. After inclusion of socio-economic deprivation in the model through the IMD, the corrected increase in ASMR decreased from to 26,9% [95% CI: 17,1 – 36,5%]% (Fig. 11).

## Discussion

There are clear spatial patterns in the distribution of environmental stress in Belgium. In all cluster categories, (air pollution clusters, land cover clusters and noise clusters), we observed clusters presenting low exposure and clusters presenting high exposure to environmental stress. At the same time, the clusters showing expected and observed unfavourable human health conditions, such as elevated levels of PM_2.5_ and NO_2_ and lack of available residential green spaces, are limited in geographical extent, these are often densely populated areas affecting a considerable share of the population. Findings of our ecological regression model are generally in line with hypotheses based on the existing literature: increased (observed > expected) ASMR rates due to exposure to high levels of air pollution, increased ASMR in traffic-associated hotspots, increased ASMR due to exposure to industrial substances other than the traditional air pollutants, increased ASMR in case of presence of primary and secondary roads. Concerning noise, we could not find any negative effects on human health, the elevated levels of railway noise even had a protective effect. We also demonstrated that cumulative exposure to multiple environmental stressors has potentially synergistic negative effects on human health.

Within the air pollution clusters, clusters B and C correspond to areas where the local air pollution component is largely caused by traffic as demonstrated by the higher NO_2_ and BC values compared to PM_2.5_. In contrast, cluster D shows similar PM_2.5_ values and lower BC, and NO_2_ values indicating that the local PM_2.5_ component may be partly originating from residential warming activities like wood burning. The increase of 14,8% [95% CI: 10,4 – 19,4%] in ASMR in the air pollution cluster C decreased to 4,9% [95% CI: 0,7 – 8,8%] after adjustments of confounding factors, which corresponds to respectively 12,8% and 4,7% attributable mortality due to air pollution differences between cluster E and C, is comparatively lower than the combined effect we can estimate based on the literature findings where we found around 12% mortality from NO_2_ and 15% from PM_2.5_ in cluster C. Considering a potential overlap of 30% [30, 34] in effects, the combined air pollution effect of NO_2_ and PM_2.5_ in Cluster C based on literature evidence is still above 20%. Moreover, the estimate from the literature originates from the ELAPSE meta-analysis, which only included studies with adjustments for socioeconomic and confounding factors.

Our ecological regression model did not reveal any significant increase in ASMR for all-cause mortality associated with exposure to high noise levels. For railway noise, we found a significant protective effect on human health. Potentially this could be confounded by the general absence of busy traffic in areas around railways, affecting human health through different pathways including air pollution, physical activity and green space availability [21].

For the land cover clusters, cluster D, presenting a high fraction of forests and green spaces, is expected to have a beneficial impact on our health and protect against mortality, as there is a negative relationship between the RR of mortality and the fraction of green space and forests within 500 m of people’s residence [25]. The significant increase in ASMR in the industrial cluster indicates that human health may be negatively impacted by industrial activities through pathways different from the typical air pollutants NO_2_ and PM_2.5_ which were explicitly considered in this study. Earlier studies hypothesized health effects from industrial activities through amongst other exposure to chemicals and toxic substances such as lead [20]. Similarly, earlier studies demonstrated that other factors than the traditional air pollutants (e.g. reduced social contacts, lack of green and recreational spaces, …) may as well be involved in explaining increased disease burden in the presence of primary and secondary roads [4, 12]. In our ecological regression model we cannot detect any observed ASMR exceeding the expected ASMR related to agriculture, implying that our study fails to detect evidence of harmful effect of proximity of agricultural land and associated practices.

In some areas, where environmental stress accumulates, synergistic effects may occur. A synergistic effect for heat and air pollution is well-established [3]. However, little research has been conducted yet and uncertainty remains important. Our ecological regression model indicates the presence of such synergistic effects. The simultaneous exposure to elevated environmental stress tends to have an increased ASMR exceeding the sum of the individual effects, which may indicate synergies. These results are crucial for policy makers in public health and the environment. The cluster analysis is usable as a tool to reduce the number of areas where human health is disproportionally affected negatively by environmental stress.

Different confounding factors such as smoking, dietary habits and alcohol intake can occult the relationship between the environmental stressors and the mortality outcomes at the ecological level. Between 10 and 47% of all-cause mortality can be attributable to smoking in Belgian municipalities [23] making it an important composite of total mortality. Adding the regions as a variable in the model reduces the influence of the confounding factor of smoking and other unaccounted confounding factors to an extent. As referenced in the methodology section, differences in smoking prevalence between the regions in Belgium are well established and recognised in existing research [23, 26]. The same is true for other behavioural confounding factors such as alcohol consumption.

Our study presents several strengths. An analysis based on the statistical sector is an approach that has a considerably higher spatial resolution compared to most existing studies, increasing the accuracy in establishing relationships between environmental stressors and all-cause mortality. A cluster analysis allows the inclusion of multiple environmental stressors at once. Few studies have examined the cumulative effects of exposure to multiple stressors. A cluster analysis approach allowed the identification and comparison of spatial patterns between the different domains under consideration. Grouping statistical sectors in clusters simplifies pattern interpretation compared to examining 20,000 individual sectors. It also facilitates the translation of research findings into relevant knowledge for policymakers in the domains of environment and public health. For example, we demonstrate that while the air pollution clusters with the worst concentrations (air pollution cluster C) cover only a small percentage of the geographical area, a considerable share of the population (20%) lives in those areas. This information is important for determining interventions to reduce mortality and improve environmental and public health outcomes. Statistically, the cluster analysis approach also provided advantages as it results in a more stable regression model because the same model including for example air pollutants like PM_2.5_ and NO_2_ as separate variables, results in severe issues of multicollinearity and therefore type I and type II errors. A cluster approach is, therefore, essential for obtaining results that allow statistical interpretation. We avoided the use of statistical methods for which it is known they can be problematic in terms of multicollinearity. While we made substantial efforts to reduce the impact of multicollinearity, a limited amount of multicollinearity can still be present despite that statistical parameters such as the Variance Inflation Factor (VIF) do not show any multicollinearity [16].

Disadvantages of the hierarchical cluster analysis approach may include loss of detail and missing outliers. It also assumes no underlying knowledge of the patterns of environmental stressors. There is always a small but real risk of exposure misclassification and inconsistent temporality of different data sets can increase uncertainties. Another limitation of our study is that it was not possible to consider all confounding factors quantitatively. Another disadvantage is that the attributable all-cause mortality for the different clusters based on existing evidence in the literature is focused on only air pollution, as no robust estimates in the literature were found for the other stressors. Additionally, this is an ecological study. Despite some innovations we used to improve performance and reliability; ecological studies are not considered robust enough for epidemiological studies. Issues of bias and fallacy cannot be excluded from being present. An inclusive perspective on all-cause mortality across various age strata, encompassing even those aged 80 and above, was adopted and can attenuate the associations between health and environmental variables. This effect arises from the incorporation of mortality factors beyond intrinsic health conditions. The integration of all causes of death, including external causes, within the context of all-cause mortality, further underscores this phenomenon. Moreover, relationships between health and both the environmental and socio-economic parameters are expected to attenuate as individuals surpass their projected lifespans, aligning with the eventual mortality of all individuals [31].

## Conclusion

The cluster analysis enabled the detection of spatial patterns of environmental stressors in Belgium. There are extensive differences in exposure to environmental stress in all considered domains – air pollution, land cover and noise – in Belgium. This has important implications for policymakers in the domains of environment and public health. With PM_2.5_ WHO target values of 5 µg/m^3^ exceeded in all clusters (100% of the population exposed) and NO_2_ target values of 10 µg/m^3^ exceeded in four of five air pollution clusters (92% of the population exposed to > 10 µg/m^3^), the WHO target value of 25% Green space only reached in one out of five land cover clusters (96% of population exposed to < 25% GA), and noise exceedances in four of five noise clusters (45% of the population exposed to > 55db from any source), the environment in Belgium does in general not qualify as healthy for our human health, with in addition the presence of large spatial variations and inequalities in exposure. The ecological regression model shows the most elevated ASMR rates in clustered areas with very high air pollution values where traffic is the predominant local air pollution component and in industrial areas, with indications industrial land affects human health through other pathways (e.g., chemical/toxic substances) in addition to the well-known air pollutants PM_2.5_ and NO_2_. As the increase in Incidence Risk Ration is higher in the cumulative model compared to the addition of the health effects in the single model, it might indicate a high probability of synergistic health effects from exposure to multiple environmental stressors at once. Ecological bias, misclassification and confounding factors can impact the quantitative estimates from the ecological regression model. However, our results are robust and show strong associations. The cluster analysis allows us to consider some areas as spatially homogenous units and, enables us to identify how to reduce inequalities in exposure to environmental stressors thereby decreasing the number of areas where human health is disproportionally affected in a negatively by environmental stress. Eventually, such an approach could support the improvement of overall population health, reduce inequalities, and cut healthcare costs such as hospital treatments and (paid) sick leave.

### Supplementary Information

**Additional file 1: Figure S1.** Characteristics of the socio-economic clusters. Average decile score for education, housing, crime and incoe for each of the socio-economic clusters. A decile score of 10 implies “lowest level of deprivation” while a decile score of 1 implies “highest level of deprivation”. **Table S1.** Negative Binomial Regression model outputs – Model without Socio-economic variables. **Table S2.** Negative Binomial Regression model outputs – Model with Socio-economic variables. **Table S3.** Negative Binomial Regression model outputs – CUMULATIVE MODEL - Model without  Socio-economic variables. **Table S4.** Negative Binomial Regression model outputs – CUMULATIVE MODEL - Model with Socio-economic variables.

## Data Availability

Environmental data was obtained through standard data request procedures at the respective environmental agencies, and was provided as geographical maps, and did not contain information on individuals. All environmental data can be directly downloaded as open data or requested from the specific data links provided below. • The air pollution data are open source and retrieved from the website of IRCEL-CELINE: https://www.irceline.be/en/documentation/open-data • The noise data was obtained through the respective environmental agencies of the different regions in Belgium: ◦ For Brussels: metadata and access request on https://datastore.brussels (more detail : https://datastore.brussels/web/data/dataset/11daaefc-2abc-4b1a-8768-4544464b452d). Those data can be requested by any organisation or any individual and no administrative permission are required to obtain or access the data. ◦ For Wallonia: metadata and access request on https://geoportail.wallonie.be/ (catalogue-donnees-et-services?search-changed=false+&search-tri=relevance&search-tab=&search-perPage=&search-text=bruit&search-diffusion-service-type=&search-not-obsolete=true&search-not-inspire=true&search-mapDate=&search-sheetDate=#results-area). Those data can be requested by any organisation or any individual and no administrative permission are required to obtain or access the data. ◦ For Flanders: The data are directly downloadable through https://www.geopunt.be • Download:https://www.vlaanderen.be/datavindplaats/catalogus/wfs-publieke-download-service-van-vlaamse-overheid-beleidsdomein-omgeving-samenwerkingsverband-mercatornet • Visualisation : https://www.vlaanderen.be/datavindplaats/catalogus/wms-publieke-view-service-van-vlaamse-overheid-beleidsdomein-omgeving-samenwerkingsverband-mercatornet • The landcover data are open source and downloaded from the CORINE Land Cover website. https://land.copernicus.eu/pan-european/corine-land-cover • Population data used are open source available from the open-data portal of Statbel. https://statbel.fgov.be/en/themes/population • Data on the statistical sectors can be downloaded from the Statbel portal. https://statbel.fgov.be/en/open-data/statistical-sectors-2021 Further, aggregated health (all-cause mortality) data was obtained via request procedure. The Belgian statistical office (Statbel) provided us with aggregated mortality data at the level of the statistical sectors, the smallest administrative units in Belgium. All data were anonymized before it’s use. The mortality data used in our study are part of the Causineq database that was obtained from the Belgian statistical office, Statbel, after approval by the Statistical Oversight Committee of the Privacy Commission. The confidentiality contract numbers are STAT-MA-2015–13 and STAT-MA-2016–23. On 31 May 2021 (decision no. 2021/071), the authors obtained the right to update, use, and store the Causineq data until 31 December 2034. The Causineq database is pseudonymised and contains a multi-digit code specific to this database. It therefore does not enable linkages with other administrative databases or databases belonging to other research centres. The data we received based on this dataset for our study were anonymised before it’s use. More specifically, data are aggregated and numbers lower than 5 were replaced by ‘NA’. No data was generated in this study. The data for analysis was obtained from third parties, for which the authors do not have the licence for further distribution. More info: https://statbel.fgov.be/en/about-statbel/what-we-do/microdata-research
