# Supplementary material for: Cluster pattern analysis of environmental stressors and quantifying their impact on all-cause mortality in Belgium
Source: BMC Public Health. 2024 Feb 21;24:536. doi: 10.1186/s12889-024-18011-0 (PMC11218127; doi:10.1186/s12889-024-18011-0)
Supplement: Supplementary file 1 — Additional file 1: Figure S1. Characteristics of the socio-economic clusters. Average decile score for education, housing, crime and incoe for each of the socio-economic clusters. A decile score of 10 implies “lowest level of deprivation” while a decile score of 1 implies “highest level of deprivation”. Table S1. Negative Binomial Regression model outputs – Model without Socio-economic variables. Table S2. Negative Binomial Regression model outputs – Model with Socio-economic variables. Table S3. Negative Binomial Regression model outputs – CUMULATIVE MODEL - Model without Socio-economic variables. Table S4. Negative Binomial Regression model outputs – CUMULATIVE MODEL - Model with Socio-economic variables. [file 12889_2024_18011_MOESM1_ESM.docx]

Additional file 1


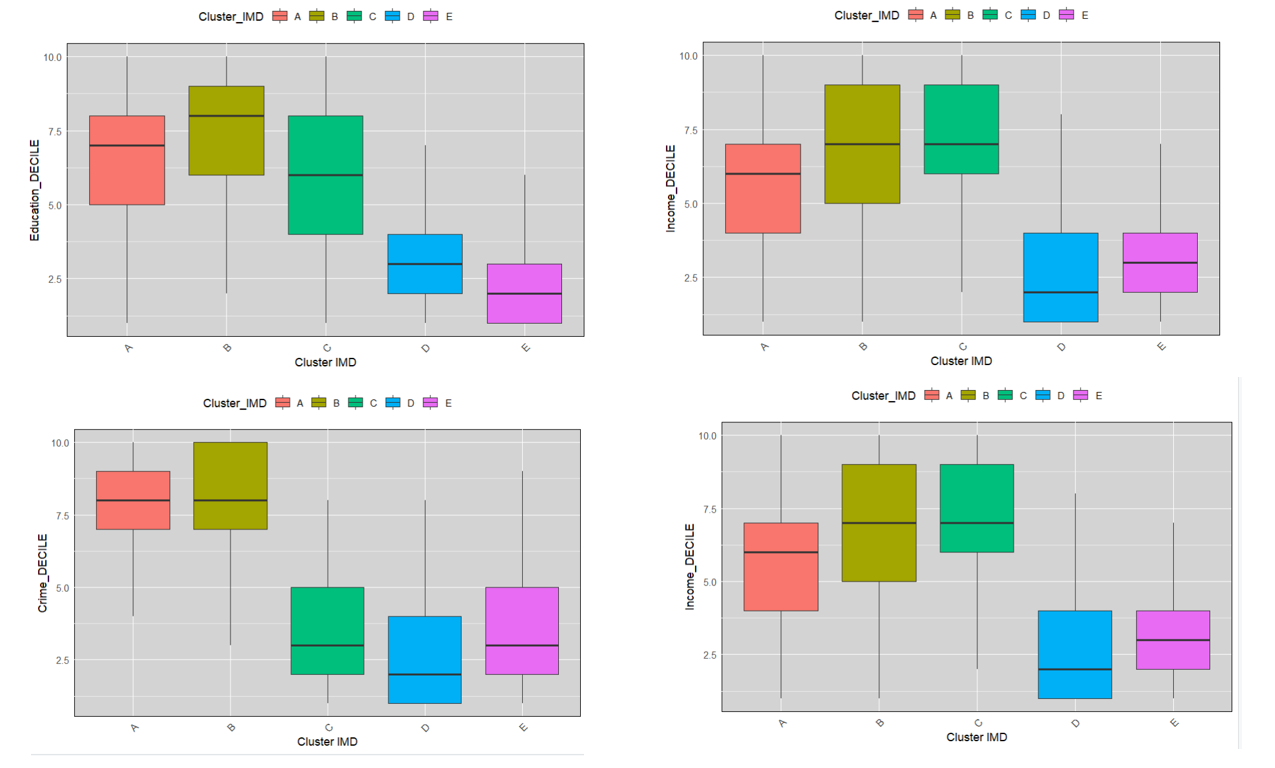


***Figure S1:*** Characteristics of the socio-economic clusters. Average decile score for education, housing, crime and incoe for each of the socio-economic clusters. A decile score of 10 implies “lowest level of deprivation” while a decile score of 1 implies “highest level of deprivation”

***Table S1:*** Negative Binomial Regression model outputs – Model without Socio-economic variables

| **Variable** | **Estimate** | **Std. Error** | **p-value** | **95% CI (z=1.96)** |
| --- | --- | --- | --- | --- |
| (Intercept) | -0.1506 | 0.0265 | - | (-0.203, -0.098) |
| CLUSTERS_AIR_POLLUTION_A | 0.0423 | 0.0119 | 0.0003 | (0.019, 0.065) |
| CLUSTERS_AIR_POLLUTION_B | 0.0552 | 0.0141 | 0.0008 | (0.028, 0.083) |
| CLUSTERS_AIR_POLLUTION_C | 0.1381 | 0.0202 | < 0.0001 | (0.099, 0.178) |
| CLUSTERS_AIR_POLLUTION_D | 0.0377 | 0.0156 | 0.0159 | (0.007, 0.068) |
| CLUSTERS_AIR_POLLUTION_E | Reference |  |  |  |
| NOISE_CLUSTER_A | -0.0277 | 0.0123 | 0.0244 | (-0.052, -0.004) |
| NOISE_CLUSTER_B | -0.0526 | 0.0339 | 0.1215 | (-0.119, 0.014) |
| NOISE_CLUSTER_C | -0.0402 | 0.0162 | 0.0129 | (-0.066, -0.014) |
| NOISE_CLUSTER_D | -0.0038 | 0.0096 | 0.6953 | (-0.017, 0.010) |
| NOISE_CLUSTER_E | Reference |  |  |  |
| REGIONBRUSSELS | Reference |  |  |  |
| REGIONFLANDERS | -0.0854 | 0.0195 | 0.0001 | (-0.123, -0.047) |
| REGIONWALLONIA | 0.1592 | 0.0218 | < 0.0001 | (0.116, 0.202) |

***Table S2:*** Negative Binomial Regression model outputs – Model with Socio-economic variables

| **Variable** | **Estimate** | **Std. Error** | **p-value** | **95% CI** |
| --- | --- | --- | --- | --- |
| (Intercept) | -0.152 | 0.027 | 0.000 | (-0.203, -0.101) |
| CLUSTERS_AIR_POLLUTION_A | 0.025 | 0.012 | 0.038 | (0.001, 0.049) |
| CLUSTERS_AIR_POLLUTION_B | 0.009 | 0.014 | 0.695 | (-0.004, 0.022) |
| CLUSTERS_AIR_POLLUTION_C | 0.048 | 0.021 | 0.022 | (0.007, 0.089) |
| CLUSTERS_AIR_POLLUTION_D | 0.014 | 0.016 | 0.920 | (-0.017, 0.045) |
| CLUSTERS_AIR_POLLUTION_E | Reference |  |  |  |
| NOISE_CLUSTER_A | -0.014 | 0.012 | 0.115 | (-0.038, 0.010) |
| NOISE_CLUSTER_B | -0.039 | 0.034 | 0.116 | (-0.076, 0.002) |
| NOISE_CLUSTER_C | -0.044 | 0.016 | 0.275 | (-0.075, -0.013) |
| NOISE_CLUSTER_D | -0.001 | 0.009 | 0.089 | (-0.010, 0.009) |
| NOISE_CLUSTER_E | Reference |  |  |  |
| LANDCOVER_CLUSTER_A | 0.060 | 0.015 | 0.000 | (0.030, 0.088) |
| LANDCOVER_CLUSTER_B | 0.043 | 0.016 | 0.007 | (0.012, 0.074) |
| LANDCOVER_CLUSTER_C | 0.110 | 0.024 | 0.000 | (0.063, 0.157) |
| LANDCOVER_CLUSTER_D | Reference |  |  |  |
| LANDCOVER_CLUSTER_E | 0.015 | 0.016 | 0.930 | (-0.016, 0.046) |
| cluster_IMD_A | -0.069 | 0.011 | 0.000 | (-0.088, -0.049) |
| cluster_IMD_B | Reference |  |  |  |
| cluster_IMD_C | -0.023 | 0.010 | 0.021 | (-0.043, -0.003) |
| cluster_IMD_D | 0.098 | 0.015 | 0.000 | (0.069, 1.265) |
| cluster_IMD_E | 0.137 | 0.011 | 0.000 | (0.116, 0.159) |
| REGIONBRUSSELS | Reference |  |  |  |
| REGIONFLANDERS | -0.047 | 0.020 | 0.017 | (-0.085, -0.007) |
| REGIONWALLONIA | 0.132 | 0.022 | 0.000 | (0.089, 0.175) |

***Table S3:*** Negative Binomial Regression model outputs – CUMULATIVE MODEL - Model without Socio-economic variables

| **Variable** | **Estimate** | **Std. Error** | **p-value** | **95% CI** |
| --- | --- | --- | --- | --- |
| (Intercept) | -0.196 | 0.030 | 0.000 | (-0.255, -0.137) |
| CUMULATIVE_LOW | Reference |  |  |  |
| CUMULATIVE_HIGH | 0.293 | 0.038 | 0.000 | (0.217, 0.369) |
| CUMULATIVE_MEDIUM | 0.214 | 0.025 | 0.000 | (0.164, 0.264) |
| REGIONBRUSSELS | Reference |  |  |  |
| REGIONFLANDERS | -0.153 | 0.017 | 0.000 | (-0.186, -0.120) |
| REGIONWALLONIA | 0.079 | 0.017 | 0.000 | (0.044, 0.114) |

***Table S4:*** Negative Binomial Regression model outputs – CUMULATIVE MODEL - Model with Socio-economic variables

| Variable | Estimate | Std. Error | p-value | 95% CI |
| --- | --- | --- | --- | --- |
| (Intercept) | -0.273 | 0.031 | 0.000 | (-0.334, -0.212) |
| CUMULATIVE_LOW | Reference |  |  |  |
| CUMULATIVE_HIGH | 0.238 | 0.037 | 0.000 | (0.165, 0.311) |
| CUMULATIVE_MEDIUM | 0.192 | 0.025 | 0.000 | (0.143, 0.241) |
| cluster_IMD_A | -0.067 | 0.012 | 0.000 | (-0.081, -0.053) |
| Cluster_IMD_B | Reference |  |  |  |
| cluster_IMD_C | -0.021 | 0.010 | 0.032 | (-0.031, -0.011) |
| cluster_IMD_D | 0.108 | 0.014 | 0.000 | (0.080, 0.136) |
| cluster_IMD_E | 0.147 | 0.010 | 0.000 | (0.127, 0.167) |
| REGIONFLANDERS | -0.064 | 0.018 | 0.000 | (-0.086, -0.042) |
| REGIONWALLONIA | 0.119 | 0.018 | 0.000 | (0.093, 0.145) |
